# Estimating Chikungunya prevalence in La Réunion Island outbreak by serosurveys: Two methods for two critical times of the epidemic

**DOI:** 10.1186/1471-2334-8-99

**Published:** 2008-07-28

**Authors:** Patrick Gérardin, Vanina Guernier, Joëlle Perrau, Adrian Fianu, Karin Le Roux, Philippe Grivard, Alain Michault, Xavier de Lamballerie, Antoine Flahault, François Favier

**Affiliations:** 1 Centre d'Investigation Clinique – Épidémiologie Clinique (CIC – EC) de La Réunion (Institut National de la Santé et de la Recherche Médicale/Centre Hospitalier Départemental – Groupe Hospitalier Sud Réunion/Union Régionale des Médecins Libéraux de la Réunion), Groupe Hospitalier Sud Réunion, BP 350, 97448 Saint Pierre cedex, La Réunion; 2 Unité de Recherche 707, Institut National de la Santé et de la Recherche Médicale, Faculté de Médecine Saint – Antoine, 27 rue de Chaligny, 75012 Paris, France; 3 Service de Bactériologie-Parasitologie-Virologie et Hygiène, Groupe Hospitalier Sud Réunion, BP 350, 97448 Saint Pierre cedex, La Réunion; 4 Unité des Virus Emergents, Faculté de Médecine, 27 bd Jean Moulin, 13005 Marseille, France; 5 École des Hautes Études en Santé Publique, avenue Léon Bernard, 35000 Rennes, France

## Abstract

**Background:**

Chikungunya virus (CHIKV) caused a major two-wave seventeen-month-long outbreak in La Réunion Island in 2005–2006. The aim of this study was to refine clinical estimates provided by a regional surveillance-system using a two-stage serological assessment as gold standard.

**Methods:**

Two serosurveys were implemented: first, a rapid survey using stored sera of pregnant women, in order to assess the attack rate at the epidemic upsurge (s1, February 2006; n = 888); second, a population-based survey among a random sample of the community, to assess the herd immunity in the post-epidemic era (s2, October 2006; n = 2442). Sera were screened for anti-CHIKV specific antibodies (IgM and IgG in s1, IgG only in s2) using enzyme-linked immunosorbent assays. Seroprevalence rates were compared to clinical estimates of attack rates.

**Results:**

In s1, 18.2% of the pregnant women were tested positive for CHIKV specific antibodies (13.8% for both IgM and IgG, 4.3% for IgM, 0.1% for IgG only) which provided a congruent estimate with the 16.5% attack rate calculated from the surveillance-system. In s2, the seroprevalence in community was estimated to 38.2% (95% CI, 35.9 to 40.6%). Extrapolations of seroprevalence rates led to estimate, at 143,000 and at 300,000 (95% CI, 283,000 to 320,000), the number of people infected in s1 and in s2, respectively. In comparison, the surveillance-system estimated at 130,000 and 266,000 the number of people infected for the same periods.

**Conclusion:**

A rapid serosurvey in pregnant women can be helpful to assess the attack rate when large seroprevalence studies cannot be done. On the other hand, a population-based serosurvey is useful to refine the estimate when clinical diagnosis underestimates it. Our findings give valuable insights to assess the herd immunity along the course of epidemics.

## Background

**Figure 1:**
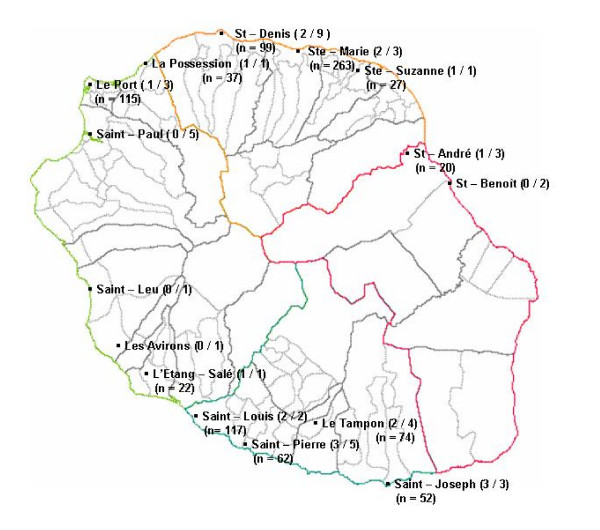
**Map of La Réunion Island**. The territory is divided into four regions: north bounded by orange and red lines, west bounded by orange, light green, dark green and red lines, south bounded by dark green and red lines, east by red lines. For each municipality, the ratio of laboratories which participated to the survey on pregnant woman and the related amount of the sera collected (n = x) are listed in parentheses.

Chikungunya fever is an arbovirosis caused by Chikungunya virus (CHIKV), a mosquito-transmitted alphavirus belonging to the *Togaviridae *family [1,2]. CHIKV was first isolated in 1952, during a Tanzanian outbreak [3]. It circulated in Africa and Asia, where periodic outbreaks were described in the past 50 years. In some areas, attack rates had reached 80 to 90% [1,2]. Between February 2005 and August 2006, a large Chikungunya fever outbreak swept the Indian Ocean islands [4,5], including La Réunion Island since April 2005, an overseas French department of 787,836 inhabitants (Figure 1). The mosquito specie involved in La Réunion outbreak was *Aedes (A.) albopictus *[6]. Most CHIKV infections were symptomatic [7] and characterized by a dengue-like illness of sudden onset combining high fever, poly-arthralgia, myalgia, headache, asthenia and rash [8,9].

In La Réunion, the epidemic pattern was monitored through a regional surveillance-system managed by the Cellule Interrégionale d'Epidémiologie (CIRE) based on "suspected cases", defined as subjects with a sudden fever (T > 38.5°C) and incapacitating arthralgia [10,11]. This surveillance-system relied on self-reports, emergency stays, physician declarations, biology laboratories activity, and active case-finding around the cases reported by a sentinel physician network [11]. At the beginning of the outbreak it consisted in an active and retrospective case detection around the cases declared, and then, when the incidence sharply increased (by December 2005), in an estimation of the cases obtained from reports of a sentinel network [12].

**Figure 2:**
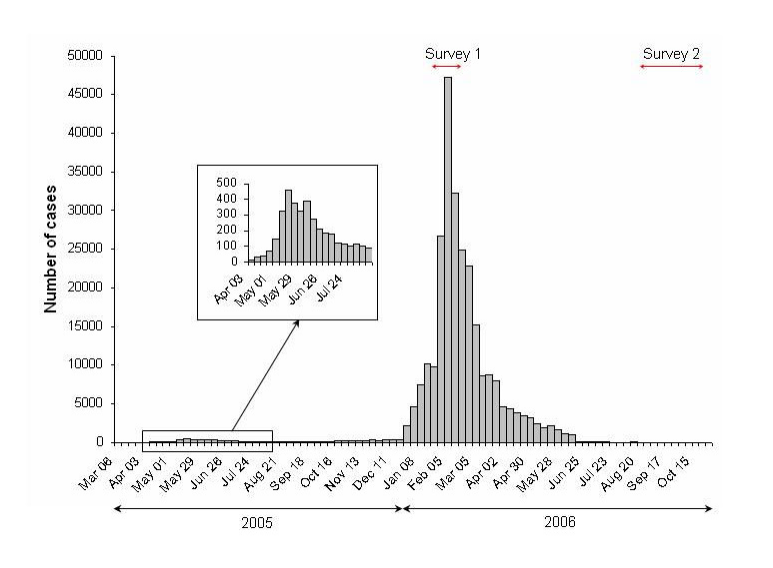
**Number of weekly incident cases of Chikungunya, La Réunion Island, March 28^th^, 2005 – April 16^th^, 2006 (n = 244,000)**. Reported by the active case-finding system between weeks 9 and 50, 2005 or estimated from the sentinel physician network between week 51 of 2005 and week 15, 2006. Published by Renault P, et al. in *Am J Trop Med Hyg*, 2007, 77: 727–731 [11], and reprinted with the kind permission of the American Society of Tropical Medicine and Hygiene (Atlanta, USA). "Survey 1" corresponds to the rapid serological survey on pregnant women (January 15^th ^2006 to February 15^th ^2006); "Survey 2" corresponds to the population-based SEROCHIK survey (August 17^th ^to October 20^th ^2006).

Before the explosion of the epidemic in mid-January 2006, a herald wave occurred during the previous rainy season; between March and July 2005, and led the CIRE to record about 3,000 suspected cases of Chikungunya [13]. Later on and until December 2005, low case rates were recorded without interruption. An exponential increase of the cases reported was observed in late December 2005, and January 2006 with a peak in February 2006 [11] (Figure 2). On February 15^th ^2006, the CIRE estimated 157,000 suspected cases of Chikungunya, *i.e*. a prevalence rate of 20.3%. On July 5^th ^2006, the CIRE estimated the burden more than 266,000, *i.e*. a prevalence rate of 34.3% [14].

The purpose of the study was to refine the estimates of attack rates provided by the surveillance-system for the population of La Réunion Island at two critical times of the 2005–2006 outbreak. That is why we conducted two serosurveys, the first using stored sera of pregnant women during the epidemic upsurge aimed at assessing the extent of the outbreak, the second using a random sample of the population aimed at giving a precise idea of the herd immunity in the post-epidemic era.

## Methods

### Rapid survey on pregnant women, epidemic phase

We gathered sera of pregnant women yet available in outpatient laboratories from a mandatory monthly serological screening for congenital toxoplasmosis. The sera, collected between January 15^th ^and February 15^th ^2006, were neither directly nor indirectly nominative, and could only be identified by a unique code number. All of the 46 biological labs of La Réunion Island were invited to participate to the survey. Out of these, the 28 participating ones served the entire territory (Figure 1). However, only 19 labs provided valid sera which led to a non-representative amount of 888 valid sera, taken out of the 3888 sera collected during the study period (389 in the north, 305 in the south, 174 in the west, 20 in the east). For this study, designed to inform without delay public health authorities on the extent of the outbreak, a selection bias related to the absence of randomisation of labs was tolerated. Nevertheless, as daily routine sampling of pregnant women was not dedicated to a precise laboratory, it is reasonable to think that the repartition bias was not significant. Statistical analysis was performed using SAS software version 8 (SAS Institute, Cary, NC).

### Population-based survey, post-epidemic phase

A cross-sectional study, the SEROCHIK survey, was conducted between August 17^th ^and October 20^th ^2006 from a random sample of the community of Reunion Island [7]. At the first sampling stage, the French National Institute for Statistics and Economical Studies (INSEE) randomly selected 3032 households after stratification on age, gender, the geographical area, municipality size, and type of habitat. The geographical area of habitat was defined according to the regional administrative boundary into four micro-regions (Figure 1). The municipality size was divided in ≤ or > 10,000 inhabitants. The type of habitat was categorized into individual or collective housing (multifamily ≤ 20 or > 20 housings). At the second stage, a Kish method was used to randomly choose one person for each selected household [13].

Of the 3032 households randomly selected by INSEE, the sampling plan led to a set of 2442 eligible subjects (after exclusion of absents, persons with invalid address or who refused to participate) which was recovered by INSEE on age, gender, geographical area, and type of habitat.

The study was approved by the ethical committee for studies with human subjects (CPP) of Bordeaux and the National Commission for Informatics and Liberty (CNIL). All participants provided their informed consent to answer the questionnaire and for collection of blood on filter paper.

Statistical analysis was done by accounting for the sampling design, and was performed using Stata software (College Station, Texas). The population-based seroprevalence was compared to CIRE clinical estimates using a Chi square test. A *P-value *< 0.05 was considered significant. The population size used for the calculation of incidence was 787,836 inhabitants (INSEE, April 2006).

### Detection of chikungunya infections

For the rapid survey in pregnant women, 100 microliters of stored sera already available in outpatient laboratories were used. In the SEROCHIK survey, for each person consenting to a fingertip prick, a drop of blood was deposited onto Whatman no.1 filter paper [15]. For both studies, anti-CHIKV specific antibodies were screened using the same enzyme-linked immunosorbent assay (ELISA) and a CHIKV antigen produced by the Centre National de Référence pour les Arbovirus (CNR, Lyon, France) [15]. For the rapid survey, the ELISA was performed at the CNR whereas for the SEROCHIK survey, it was done using the Groupe Hospitalier Sud – Réunion (GHSR) laboratory facilities. Both IgM and IgG anti-CHIKV specific antibodies were screened in sera from pregnant women, whereas only IgG anti-CHIKV specific antibodies were screened in the community. In parallel with the SEROCHIK survey, the ability of the prick-method to discriminate the serological status was validated in an independent sample (Fianu et al, unpublished data).

## Results

The Chikungunya serological status (positive/negative) stratified for each study is given in Table 1.

**Table 1:** Chikungunya serological status during the epidemic upsurge phase (rapid survey) and the post-epidemic era (population-based survey), Reunion Island outbreak, 2005 – 2006.

Study	Negative serology		Positive serology		Total
			
Pregnant women (rapid survey)	726	(81.8)	162	(18.2) ^†^	888
Population (population – based survey)	1475	(60.4)	967	(39.6) ^‡^	2442

Data are numbers of persons questioned and (row) percentages.

^† ^IgM and IgG anti-ChikV specific antibodies tested.

^‡ ^IgG anti-ChikV specific antibodies tested.

### Survey on pregnant women, epidemic phase

During the studied period (epidemic phase, Figure 2), 162 pregnant women (out of 888 enrolled, *i.e*. 18.2%) tested positive for antibodies to CHIKV (IgM and/or IgG). There was serological evidence for a recent Chikungunya infection, as 123 subjects (13.8%) showed both IgM and IgG, and 38 (4.3%) had IgM in the absence of IgG. Isolated positive IgG were detected in only one case (0.1%).

**Figure 3:**
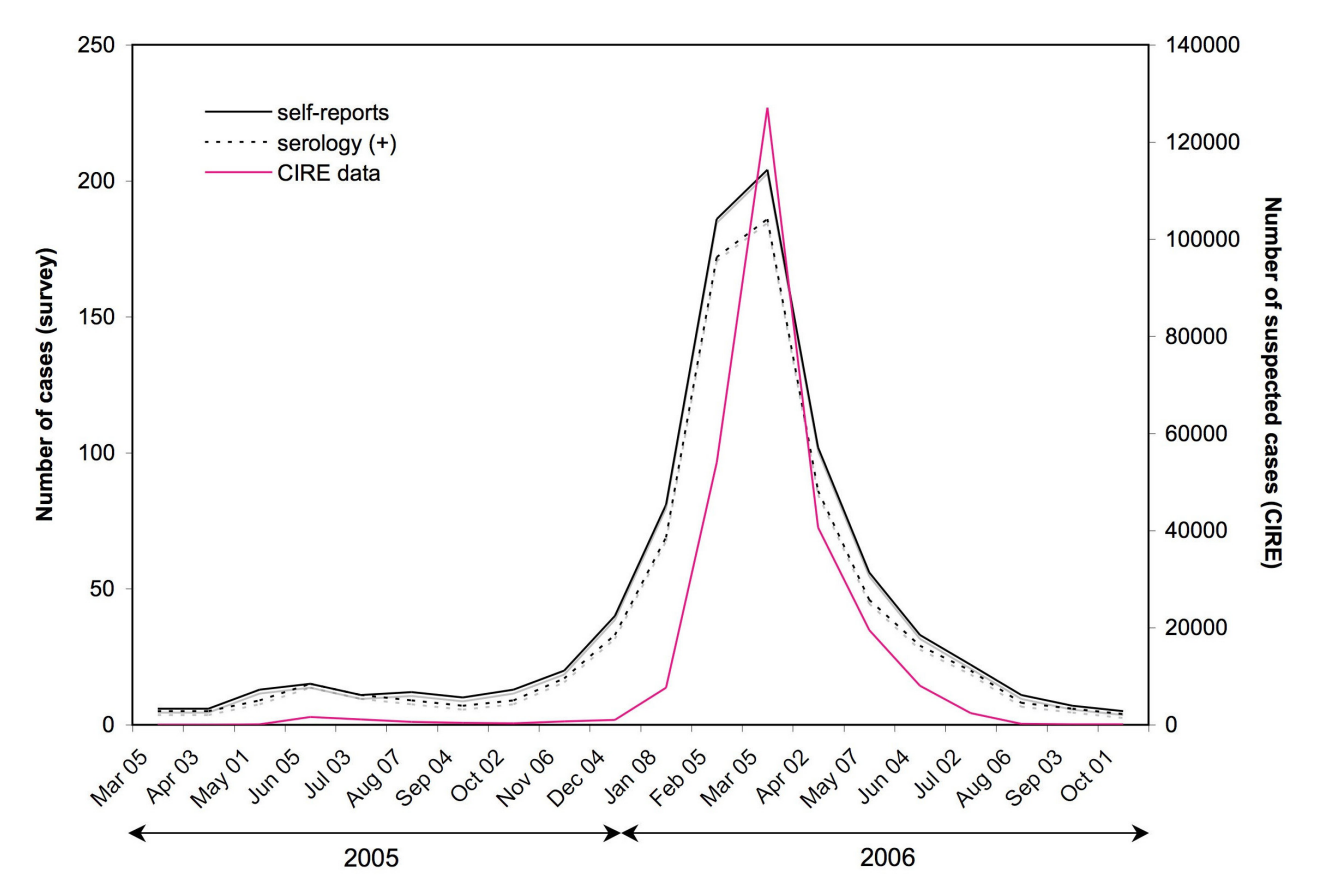
**Comparison of monthly suspected, self-reported and confirmed cases of Chikungunya in La Réunion Island between April 2005 and October 2006**. The number of suspected cases recorded weekly by the CIRE (left scale) is compared to the number of cases identified in the population-based SEROCHIK survey (right scale). For the serosurvey, both self-reports (all subjects who have declared that they have been infected, without taking into account serology results) and confirmed self-reports (with a positive serology) are noted. We refer to the date of first clinical signs declared by the subjects during the survey conducted between August 17^th ^2006 and October 20^th ^2006. "Suspected cases" are defined as cases with a sudden onset of fever with temperature > 38.5°C and incapacitating arthralgia.

Application of the Chikungunya prevalence obtained from the pregnant women group to the community resulted in a rough estimate of 143,386 (787,836 × 0.182) infected cases in La Réunion Island by February 15^th ^2006 (Figure 3).

### Population-based survey, post-epidemic phase

The seroprevalence result in the sample questioned is presented in Table 2. The overall seroprevalence for the community after recovery by the sampling plan weight (data not shown) was estimated at 38.2% (95% CI, 35.9 to 40.6%), *i.e*., about 300,000 people infected (95% CI, 283,000 to 320,000) which was significantly different that the 34.3% of people infected reported by CIRE (Chi square test: 16.4, *P *< 0.001). The rate of inapparent cases combined to atypical cases (*i.e*., all subjects who were not aware of their infection but were tested serologically positive to CHIKV) was calculated to be 5.0% (95% CI 3.9 – 6.2%), and the rate of false positive (*i.e*., subjects who declared that they were infected but had negative serology to CHIKV) was estimated to be 4.5%% (95% CI 3.7 – 5.6%). Out of the 967 serologically positive persons, 162 (16.7%) reported no symptoms.

**Table 2:** Chikungunya clinical status × serological status in the community.

**Population-based survey, Reunion Island outbreak, August – October 2006 (post-epidemic era)**						
Chikungunya declared	Negative serology		Positive serology		Total	
			
"No"	[82.5]	1217	[12.0]	116	[54.5]	1333
		(91.3)		(8.7)		(100)
Yes"	[8.0]	118	[83.2]	805	[37.9]	923
		(12.8)		(87.2)		(100)
"I don't know"	[9.5]	140	[4.8]	46	[7.6]	186
		(75.3)		(24.7)		(100)
Total	[100]	1475	[100]	967	[100]	2442
		(60.4)		(39.6)		(100)

Data are numbers of persons questioned and (row) or [column] percentages.

As the CIRE only considered suspected cases, *i.e*. subjects with fever above 38.5°C with incapacitating arthralgia, we also took into account the symptoms reported in the questionnaire of the SEROCHIK population-based survey.

The ten most frequent symptoms self-reported by the subjects who declared a Chikungunya are presented with their positive predictive value (PPV) in order of decreasing frequency in Table 3. People who declared a Chikungunya with sudden fever and incapacitating arthralgia represented 88.5% of all people who declared the disease and 87.0% of all positive serology (sensitivity and PPV for both signs: 88.5% and 87.0%, respectively).

**Table 3:** Chikungunya self-reported symptoms × serological status in the community.

**Population-based survey, Reunion Island outbreak, August – October 2006 (post-epidemic era)**					
Symptoms	Negative serology		Positive serology *		Total
			
Arthralgia	111	(12.6)	770	(87.4)	881
Fever	92	(10.9)	753	(89.1)	845
Asthenia	89	(12.8)	605	(87.2)	694
Headache	69	(11.2)	545	(88.8)	614
Myalgia	68	(12.0)	497	(88.0)	565
Rash	41	(7.7)	493	(92.3)	534
Pruritus	36	(7.9)	421	(92.1)	457
Vomiting	31	(14.2)	188	(85.8)	219
Diarrhea	21	(13.5)	135	(86.5)	156
Depression	19	(13.2)	125	(86.8)	144
Fever and arthralgia	89	(13.0)	594	(87.0)	683
Total	118	(100)	805	(100)	923

Data are numbers of persons questioned and (row) percentages. Positive Predictive value (PPV) *

## Discussion

In the current paper, we demonstrate the usefulness of two different epidemiological methods to assess the burden of a Chikungunya outbreak at two critical times of its evolution.

The rapid seroprevalence survey conducted at the peak of La Réunion Island epidemic on sera from pregnant women provided an 18.2% seroprevalence rate (or a rough estimate of 143,000 people infected) by February 15^th^, 2006. This result was obtained at a very low cost, at a time when the attack rate of the infection in the population and the herd immunity were unknown. Since 99% of the 162 tested sera harbored IgM anti-CHIKV antibodies and only one IgG anti-CHIKV antibodies only, it excluded a previous (recent) significant circulation of the virus in the island and thus suggested that the outbreak emerged into a naïve population. This result is in agreement with the 20% prevalence rate calculated for all parturient women delivered at the GHSR maternity in mid-February 2006 [16], and with the magnitude of 26% reported in pregnant women by April 2006 in Mayotte [17].

The rough estimate by the rapid serosurvey in pregnant women is slightly higher but of the same magnitude than the 130,000 cumulative number of suspected cases deducted from the CIRE at the same time (attack rate of 16.5%) [18,19]. It is therefore noteworthy that the rate observed in a targeted population of pregnant woman gives a valuable insight upon the magnitude of the attack rate in the community, and beyond, of the herd immunity. This congruent result with the CIRE data suggests that in February 2006 pregnant women yet behaved like everyone and that their level of exposure to CHIKV was not different to that observed for anybody else. In other words, the message aimed at crystallising the pregnant woman as vulnerable person to the threat of Chikungunya [20] and the measures aimed at reducing her exposure [21], *e.g*., wear of long clothing, free distribution of insecticide-treated nets and repellents, soon implemented in the maternities of La Réunion, were not effective against *A. albopictus *bites, a vector which was proven to exercise a diurnal activity [6].

The slight difference between pregnant women and community may account for a selection phenomenon, the pregnant women being not representative of the community. However, the prevalence was higher in pregnancy which argues against a significant selection bias, since more than 50% of serum collections came from south and west labs at a time when transmission was still predominant in theses micro-regions, leading to an unexpected geographical adjustment on the transmission level.

The population-based survey provided a 38.2% prevalence rate in the post-epidemic era, *i.e*., about 300,000 people infected, by October 20, 2006 [7]. At the same time, the CIRE data, published on the *Institut national de Veille Sanitaire *website, estimated at 266,000 (34.3%) the number of people infected [14]. Thus, the seroprevalence in the community was slightly higher than the 34.3% estimated for suspected cases (*P *< 0.001), but of the same magnitude. This difference might correspond to undeclared cases to the CIRE, *i.e*., (1) the inapparent cases (5.0%), although these would be compensated by an approximately equal proportion of false positives (4.5%); (2) patients who did not consult and performed auto-medication; (3) patients who did not match the clinical criteria for "suspected cases", *i.e*., sudden fever > 38.5 C° and incapacitating arthralgia. Based on laboratory confirmations of atypical presentations, Renault *et al*. calculated that approximately 3% of patients did not fulfil this definition [11]. Importantly, the SEROCHIK survey showed that 25.8% of CHIKV-infected subjects did not declare fever combined to arthralgia. This significant discrepancy may result both from an information bias due to the structure of the questionnaire (subjects who were not aware of their infection did not answer to questions about clinical signs), or a memory bias due to the time interval between the SEROCHIK survey and the onset of symptoms (2 to 15 months) that could preclude mild cases to remind their symptoms. However, the declaration-based surveillance system based on suspected cases may have also underestimated the attack rate at the epidemic phase. The difference was particularly notable from April to December 2005 (Figure 3), when the incidence was less than 500 new cases per week (Figure 2) and the surveillance relied upon active and retrospective case detection around the cases declared. Ditto, it was verified from June 2006 (Figure 3), when the incidence dropped dramatically shortly before the epidemic stopped in August (Figure 2). It could be explained by a lower PPV for each symptoms (< 85 to 92%) and for the clinical definition (< 87%), as the transmission was low (Table 3). Indeed, it is well known that when the incidence of a communicable disease is low, its contribution to the clinical forms that can evoke it decreases, with a consequent decline of the PPV for clinical signs to identify the disease [22]. Moreover, the possibility of concomitant circulation of other infections, such as Influenza or Dengue [23], may have challenged the diagnosis of Chikungunya [24], especially between April and December 2005, or from June 2006.

Another important aspect of Chikungunya disease disclosed in the course of La Réunion outbreak is the low proportion of inapparent forms (16%), in comparison with those usually observed for other arbovirosis, such as Dengue Fever (> 50%) [25,26] or West-Nile virus infection (> 70%) [27,28].

Finally, it is noteworthy that the 38.2% seroprevalence rate observed in the post-epidemic phase [7] gives a valuable insight upon the herd immunity and a clearer picture of susceptible people who could be infected in the future (62.8%). Importantly, the seroprevalence observed in La Réunion Island was far inferior to those reported recently from the Kenyan island of Lamu (75%) [29] and the Grande Comoro Island (63%) [30], two areas where CHIKV emerged before to reach La Réunion [4,5].

Several hypotheses may explain this discrepancy in prevalence rates: 1°) Kenyan and Comorian climates are less prone to seasonal variations and therefore more conducive to a sustainable transmission; 2°) *A. aegypti*, the classical vector of Chikungunya, involved in Kenyan and Comorian outbreaks, keeps a better capability to spread the disease in domestic environment than the less anthropophilic *A. albopictus*; 3°) for the same reason, the density of susceptible hosts would be less important in peridomestic environment in La Réunion, than in Kenyan and Comorian homes invaded by highly anthropophilic *A. aegypti*; 4°) in La Réunion, floods brought by the cyclone *Diwa *drove away larvae of *A. albopictus *from gullies and hastened the decrease in transmission from early March 2006; 5°) effective vector control measures combining eradication of breeding sites, adulticide and larvicide treatments contributed to limit the density of vectors throughout the Reunion outbreak; 6°) In La Réunion, the herd immunity was gained more rapidly in the littoral plains (where most of the population lives in contact with vectors) which reduced transmission as the entry in the dry austral winter. Indeed, some micro-geographical differences in prevalence rates would have been not detected by the SEROCHIK survey (whose sampling plan was aimed at discriminating between micro-regions but not within), and thereby prevalence rates in littoral plains would have far exceeded the overall 38.2% rate, which would have led to the premature decline of the epidemic due to effective herd immunity.

Prevalence rates of 60 to 70% were necessary to delay resurgences to 20 to 30 years in areas where CHIKV had circulated before [31]. According to a recent mathematical model [32], it was concluded that in the best-fitting case (reproductive number of 3.7), the attack rate would have been of 73% which suggests that, with only 38.2% of people infected, a re-emergence in La Réunion Island cannot be excluded for the next years, as long as viral strains circulate in the region. However, the scenario observed in La Réunion seems thwart this mathematical prediction, because so far, no case of Chikungunya has been scientifically confirmed since August 2006. This could be explained at municipality level by heterogeneity of reproductive numbers that would have been very sensitive to local interventions in vector control [33].

## Conclusion

Congruent estimates of Chikungunya attack rate were observed at the upsurge of La Réunion Island outbreak, either using clinical declaration of suspected cases (16.5%), or a rapid serosurvey in pregnant women (18.2%). In contrast, a discrepancy was observed in the post-epidemic era, when clinical diagnosis underestimated the attack rate (34.3%) in comparison to seroprevalence estimate (38.2%; 95% CI, 35.9 to 40.6%). Thus, a rapid serosurvey in a targeted population can be helpful to assess the extent of epidemics at time of emergency when large seroprevalence studies cannot be done. Beyond this indication, our findings suggest that prospective real time surveillance of attack rates in pregnant women would serve as a good model for population monitoring in the event of Chikungunya outbreaks. However, although it may fail to detect micro-geographical differences in prevalence rates at municipality level, a population-based serosurvey can still be useful to refine the clinical estimates and to assess more precisely the herd immunity. Moreover, only a representative survey can bring an overview on risk factors and other conditions facilitating the transmission. Finally, this work speaks to the usefulness of serosurveys for the quantification of epidemics, as to cost-containment in public health. It also provides valuables clues for monitoring epidemics in high and low-incomes countries.

## Competing interests

The authors declare that they have no competing interests.

## Authors' contributions

FF, ANF and XDL conceived and designed the experiments. KLR, PHG and AM performed the experiments including serology assays. JP designed the sampling plan for the SEROCHIK survey. JP, ADF and PAG analysed the data. VG wrote the initial draft and PAG revised the manuscript, which was extensively reviewed and approved by all authors.

## Pre-publication history

The pre-publication history for this paper can be accessed here:


